# Leucine Biosynthesis Is Involved in Regulating High Lipid Accumulation in *Yarrowia lipolytica*

**DOI:** 10.1128/mBio.00857-17

**Published:** 2017-06-20

**Authors:** Eduard J. Kerkhoven, Young-Mo Kim, Siwei Wei, Carrie D. Nicora, Thomas L. Fillmore, Samuel O. Purvine, Bobbie-Jo Webb-Robertson, Richard D. Smith, Scott E. Baker, Thomas O. Metz, Jens Nielsen

**Affiliations:** a Systems and Synthetic Biology, Department of Biology and Biological Engineering, Chalmers University of Technology, Göteborg, Sweden; b Novo Nordisk Foundation Center for Biosustainability, Chalmers University of Technology, Göteborg, Sweden; c Earth and Biological Sciences Directorate, Pacific Northwest National Laboratory, Richland, Washington, USA; d National Security Directorate, Pacific Northwest National Laboratory, Richland, Washington, USA; e Novo Nordisk Foundation Center for Biosustainability, Technical University of Denmark, Hørsholm, Denmark; Korea Advanced Institute of Science and Technology

**Keywords:** biofuels, biotechnology, metabolic engineering, systems biology, yeast

## Abstract

The yeast *Yarrowia lipolytica* is a potent accumulator of lipids, and lipogenesis in this organism can be influenced by a variety of factors, such as genetics and environmental conditions. Using a multifactorial study, we elucidated the effects of both genetic and environmental factors on regulation of lipogenesis in *Y. lipolytica* and identified how two opposite regulatory states both result in lipid accumulation. This study involved comparison of a strain overexpressing diacylglycerol acyltransferase (*DGA1*) with a control strain grown under either nitrogen or carbon limitation conditions. A strong correlation was observed between the responses on the transcript and protein levels. Combination of *DGA1* overexpression with nitrogen limitation resulted in a high level of lipid accumulation accompanied by downregulation of several amino acid biosynthetic pathways, including that of leucine in particular, and these changes were further correlated with a decrease in metabolic fluxes. This downregulation was supported by the measured decrease in the level of 2-isopropylmalate, an intermediate of leucine biosynthesis. Combining the multi-omics data with putative transcription factor binding motifs uncovered a contradictory role for TORC1 in controlling lipid accumulation, likely mediated through 2-isopropylmalate and a Leu3-like transcription factor.

## IMPORTANCE

The ubiquitous metabolism of lipids involves refined regulation, and an enriched understanding of this regulation would have wide implications. Various factors can influence lipid metabolism, including the environment and genetics. We demonstrated, using a multi-omics and multifactorial experimental setup, that multiple factors affect lipid accumulation in the yeast *Yarrowia lipolytica*. Using integrative analysis, we identified novel interactions between nutrient restriction and genetic factors involving regulators that are highly conserved among eukaryotes. Given that lipid metabolism is involved in many diseases but is also vital to the development of microbial cell factories that can provide us with sustainable fuels and oleochemicals, we envision that our report introduces foundational work to further unravel the regulation of lipid accumulation in eukaryal cells.

## INTRODUCTION

The yeast *Yarrowia lipolytica* has been identified as a promising microbial cell factory for the production of biofuels and oleochemicals (1), and while metabolic engineering approaches have been employed to increase its lipid production (2–4), how its lipid metabolism is regulated remains largely unknown. Understanding the regulation of lipid metabolism in *Y. lipolytica* is critical for the further development of this yeast into a versatile and robust microbial cell factory. Moreover, knowledge concerning the regulation of its lipid metabolism will allow further harnessing of *Y. lipolytica*’s potential by surveying the full lipogenesis landscape (5). There have been a few studies on the regulation of *Y. lipolytica* lipid metabolism during nitrogen limitation (N-*lim*) (6) that monitored transcriptional changes during a shift from biomass production to lipid accumulation (7) and that have identified roles for single regulators such as Mga2 (5), Snf1 (8), Mig1 (9), and TORC1 (4). To get a more complete picture, we set out to study regulation of *Y. lipolytica* lipogenesis at the genome level using chemostat cultures.

Similarly, it has been shown that lipid accumulation can be influenced by various factors, referred to as the lipogenesis landscape of *Y. lipolytica* (5). We focused on two categories of factors, environmental and genetic, by comparing the results of *Y. lipolytica* cultivation performed under conditions of either nitrogen or carbon limitation using two different strains: a diacylglycerol acyltransferase (DGA)-overexpressing strain with high lipid production and a control strain. *Y. lipolytica* has two DGA genes, in contrast to *Saccharomyces cerevisiae*, which has only one such gene, and both overexpression of *DGA1* and overexpression of *DGA2* increase lipid accumulation (2, 10). From transcriptome analysis of a *DGA1* overexpression strain, it has been postulated that this strain redirects carbon flux from amino acid metabolism toward lipogenesis during lipid accumulation (11). However, transcriptional responses are not necessarily translated into functional changes on the level of proteins and metabolic fluxes. We therefore set out to advance our understanding of the regulation of lipid metabolism by integrating changes in the levels of transcripts with changes in protein levels, metabolic fluxes, and metabolite concentrations. Here we identified important roles for key regulators that are highly conserved across eukaryotes.

## RESULTS

### Phenotypic changes during chemostat cultivations.

A *DGA1*-overexpressing strain and a control strain were both exposed to nitrogen or carbon limitation conditions using chemostat cultivations operated at a dilution rate of about 0.05 h^−1^, which is roughly 25% of the maximum growth rate (Table 1). For each condition, we performed biological experiments in triplicate.

**TABLE 1:** Physiological parameters

Strain	Value(s)			
	WT		DGA1	
Restriction	Carbon	Nitrogen	Carbon	Nitrogen
Specific growth rate^a^ (h^−1^)	0.048 (± 0.001)	0.049 (± 0.005)	0.047 (± 0.004)	0.048 (± 0.002)
Maximum growth rate^b^ (h^−1^)	0.191 (± 0.0045)	0.180 (± 0.0025)	0.222 (± 0.0085)	0.220 (± 0.0032)
Biomass (g liter^−1^)	1.98 (± 0.06)	2.01 (± 0.18)	2.14 (± 0.10)	2.66 (± 0.46)
Nonlipid biomass^c^ (g liter^−1^)	1.89 (± 0.06)	1.84 (± 0.22)	2.05 (± 0.10)	2.14 (± 0.42)
Extracellular glucose level (g liter^−1^)	0	18.9 (± 0.4)	0	17.9 (± 0.8)
*r*_Gluc_ (mmol gDW^−1^ h^−1^)	0.65 (± 0.01)	0.72 (± 0.07)	0.61 (± 0.06)	0.64 (± 0.06)
*r*_O2_ (mmol gDW^−1^ h^−1^)	1.7 (± 0.2)	2.1 (± 0.2)	1.3 (± 0.3)	2.1 (± 0.3)
*r*_CO2_ (mmol gDW^−1^ h^−1^)	1.67 (± 0.06)	2.1 (± 0.2)	1.5 (± 0.1)	2.2 (± 0.3)
RQ	0.97 (± 0.12)	0.97 (± 0.05)	1.15 (± 0.20)	1.0 (± 0.1)
*Y*_sx_ (gDW g glucose^−1^)	0.41 (± 0.01)	0.37 (± 0.01)	0.43 (± 0.02)	0.42 (± 0.01)

a Data represent dilution rates during the chemostat stage.

b Data were determined from the exponential phase during the batch stage.

c Data were obtained by subtracting the measured lipid concentration as depicted in Fig. S1 in the supplemental material. Data are means (SD) of results from three independent chemostats. *r*_Gluc_ (mmol gDW^−1^ h^−1^), glucose uptake rate in millimoles per gram dry weight per hour; *r*_O2_, oxygen uptake rate; *r*_CO2_, CO_2_ excretion rate; RQ, respiratory quotient; *Y*_sx_, biomass yield.

Both the genetic and environmental factors, i.e., nutrient limitation and *DGA1* overexpression, had major effects on the physiology of the yeast as determined by its lipid content (see Fig. S1 in the supplemental material), while other physiological parameters, e.g., the specific glucose uptake rate, remained largely unchanged (Table 1). Total lipid accumulation was not significantly induced by *DGA1* overexpression alone or by nitrogen limitation alone; such induction was seen only when the two factors occurred together (Fig. S1A). Several different lipid classes contributed to the total lipid accumulation, albeit *DGA1* catalyzes only the last step of triacylglycerol biosynthesis (Fig. S1B). Regardless, this supports previous findings where overexpression of *DGA1* had been identified as a promising strategy to increase lipid production in *Y. lipolytica* (2).

10.1128/mBio.00857-17.1 FIG S1  Effect of genetic and environmental factors on *Y. lipolytica* lipid levels. For comparisons, measurements from the *DGA1*-overexpressing strain were taken from reference 11. (A and B) Total lipid levels (A) and lipid levels in each class (B). SE, sterol esters; TAG, triacylglycerols; FFA, free fatty acids; ES, ergosterol; PA, phosphatidic acid; CL, cardiolipin; PE, phosphatidylethanolamine; PC, phosphatidylcholine; PS, phosphatidylserine; PI, phosphatidylinositol. The rationale for the increases in the phospholipid levels upon *DGA1* overexpression is unclear, as discussed in reference 11, but they are unlikely to be due solely to the increased size of lipid droplets upon TAG accumulation. Furthermore, phospholipids do not increase upon TAG accumulation during *DGA2* overexpression (10), supporting that TAG accumulation and phospholipid accumulation are not as tightly linked as suggested here. (C) Ratio of total fatty acid composition, after whole-cell hydrolysis. Significance levels of results of comparisons of samples were calculated with the homoscedastic *t* test. Download FIG S1, PDF file, 3.4 MB. Copyright © 2017 Kerkhoven et al. 2017 Kerkhoven et al. This content is distributed under the terms of the Creative Commons Attribution 4.0 International license.

### Transcriptional changes.

**FIG 1:**
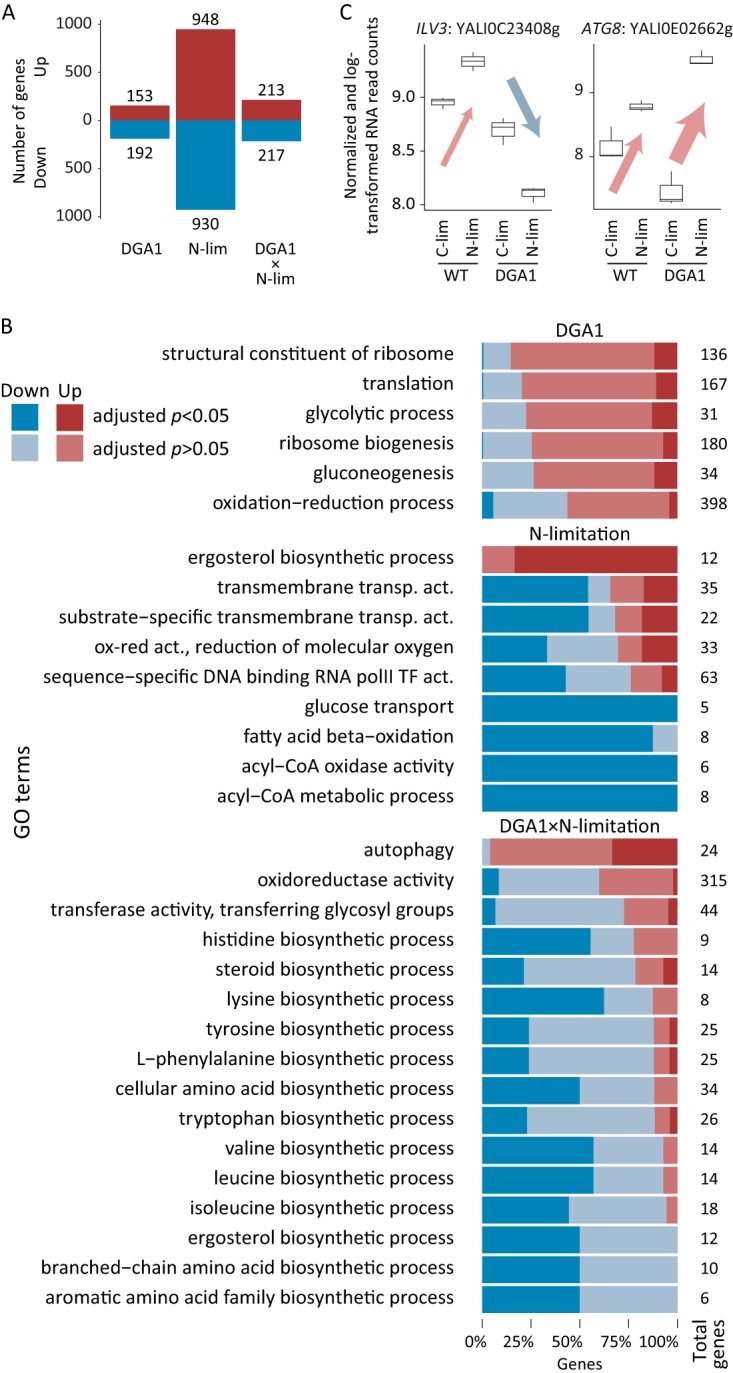
Consensus gene-set analysis using differential RNA expression according to a general linear model. (A) Overview of number of genes whose expression values were significantly (adjusted *P* < 0.05) influenced by *DGA1* overexpression (DGA1), nitrogen limitation (N-lim), or the DGA1 × N-*lim* interaction. (B) GO term enrichment analysis. For each significantly changed GO term (rank score of ≤5), the direction and significance of the changes in RNA levels of their constitutive genes are shown, together with the total number of genes within each GO term. ox-red, oxidation-reduction; polII, polymerase II; TF, transcription factor; transp. act., transporter activity. (C) Expression profiles of *ILV3*, involved in leucine biosynthesis, and *ATG8*, involved in autophagy, exemplifying the factorial effects. acyl-CoA, acyl-coenzyme A.

To establish the relative levels of the transcripts, samples were taken from the chemostat cultivations and analyzed by RNAseq, and normalized counts were used for relative quantifications. The multifactorial design of the study allowed separation of the contributions of each individual factor to the expression level. A general linear model was constructed describing the expression level based on the following factors: (i) baseline expression; (ii) expression due to *DGA1* overexpression; (iii) expression due to nitrogen limitation; and (iv) expression due to an interaction between *DGA1* overexpression and nitrogen limitation (DGA1 × N-*lim*). While nitrogen limitation had the largest effect on the differential expression of transcripts, with almost a sixth of the genes being differentially expressed (adjusted *P* < 0.05), the overexpression of *DGA1* and DGA1 × N-*lim* interaction also resulted in large transcriptional changes (Fig. 1A; see also Data Set S1 in the supplemental material).

10.1128/mBio.00857-17.8 DATA SET S1  Differentially expressed genes. An Excel file with differentially expressed genes (adjusted *P* < 0.05), as identified from the general linear model, listed per factor, is shown. For each gene, predicted GO terms are indicated, as obtained from reference 11. Download DATA SET S1, XLSX file, 0.2 MB. Copyright © 2017 Kerkhoven et al. 2017 Kerkhoven et al. This content is distributed under the terms of the Creative Commons Attribution 4.0 International license.

Gene-set enrichment analysis was performed to identify the systemic response to each of the three factors. The effect of nitrogen limitation was dominated by a downregulation of beta-oxidation, transmembrane transporter, and transcription factors (Fig. 1B), with many of the genes downregulated significantly (adjusted *P* < 0.05). These results are shown to be more coherent by examination from a different perspective: glucose limitation results in upregulation of the aforementioned gene sets. The restricted availability of carbon triggers the cells to increase expression of transmembrane glucose transporters and to scavenge carbon from storage lipids by upregulating beta-oxidation. The upregulation of ergosterol biosynthesis upon nitrogen limitation is striking. In *Y. lipolytica*, lipids accumulate in lipid droplets that contain triacylglycerol and sterol esters; however, the highest lipid accumulation was observed only when nitrogen limitation occurred in combination with *DGA1* overexpression (Fig. S1).

Overexpression of *DGA1* resulted in an upregulation of genes associated with various GO terms related to ribosomes and translation (Fig. 1B), although the enrichment of these GO terms occurs mainly through the general upward drift of their constituent genes and, to a lesser extent, as a consequence of highly significantly changing transcript levels. While these changes hint at an increased level of activity of TORC1 (12), this effect is not very strong, and it is unclear how *DGA1* overexpression would activate TORC1. In nitrogen-limited shake-flask cultivations of wild-type (WT) *Y. lipolytica* (6), the ribosome was identified as significantly downregulated, and inhibition of translation with cycloheximide resulted in moderately increased lipid accumulation. In our experiments, however, it was not *DGA1* overexpression itself that provoked the largest phenotypic change but, rather, the combination of *DGA1* overexpression and nitrogen restriction that resulted in the highest lipid accumulation (Fig. S1A).

The transcriptional changes due to the DGA1 × N-*lim* factor were dominated by an upregulation of autophagy and a downregulation of amino acid biosynthesis (Fig. 1B). The strong upregulation of autophagy genes such as *ATG8* (Fig. 1C), together with an upregulation of genes involved in allantoin degradation (Data Set S1), suggests activation by the Gln3 transcription factor (13). During nitrogen limitation, amino acid biosynthesis is upregulated, while additional overexpression of *DGA1* results in repression (Fig. 1C; Fig. S2), indicating that Gcn4 is involved in its regulation (14).

10.1128/mBio.00857-17.2 FIG S2  Transcript levels of differentially expressed cellular amino acid metabolism genes. Of the 34 genes corresponding to the GO term “cellular amino acid metabolism”, 13 were differentially expressed between the conditions (general linear model; adjusted *P* < 0.01). The normalized and log-transformed RNA read counts are shown for each of the four conditions. While nitrogen limitation in the control strain increases RNA levels, the combination of *DGA1* overexpression and nitrogen limitation represses the expression of most genes. Download FIG S2, PDF file, 3.4 MB. Copyright © 2017 Kerkhoven et al. 2017 Kerkhoven et al. This content is distributed under the terms of the Creative Commons Attribution 4.0 International license.

### Identification of transcription factor binding motifs.

**FIG 2:**
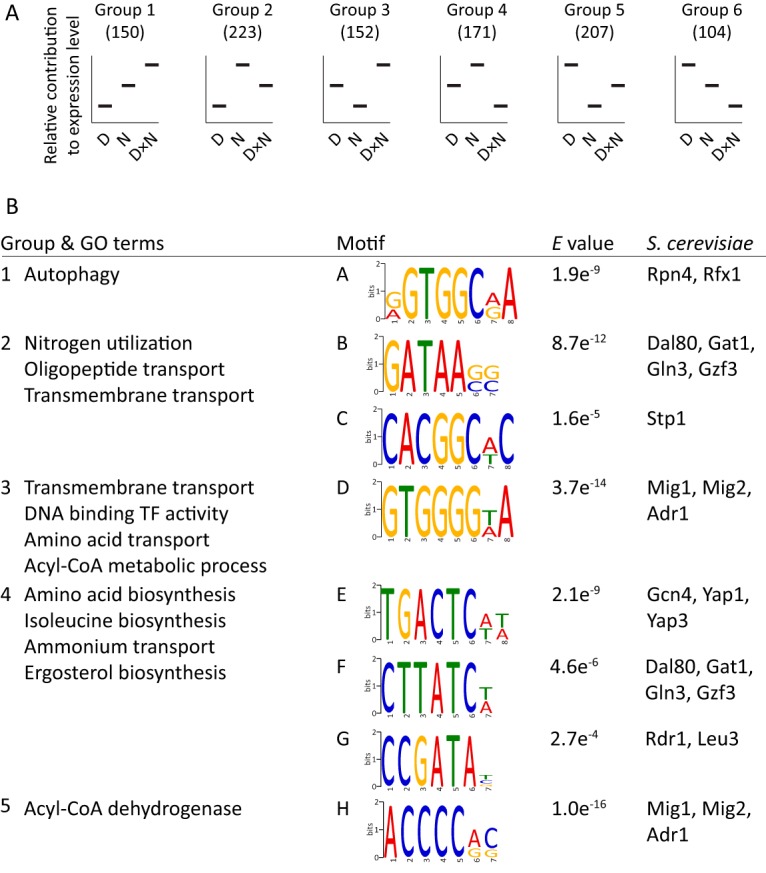
Enrichments of GO terms and transcription-factor binding motifs from genes with similar regulatory responses. (A) For each differentially expressed gene (adjusted *P* < 0.01), the relative contributions of the three factors (D, *DGA1* overexpression; N, nitrogen limitation; D×N, interaction of *DGA1* overexpression and nitrogen limitation) were ranked. The genes with the same order of factor contributions were subsequently clustered together (the number of genes per cluster is indicated). For example, for genes in group 4 (e.g., *ILV3*; see Fig. 1C), nitrogen limitation positively affects transcript levels, while D×N has a negative effect. *DGA1* overexpression by itself has an effect that is between those of the other two factors. (B) For each cluster, (i) the GO term enrichment (redundant terms removed by manual curation) and (ii) the motifs found by DREME (15), their *E* value, and *S. cerevisiae* transcription factors with similar binding motifs, as identified with Tomtom (31), are indicated.

The expression dynamics described above raised questions concerning whether common transcription factors are involved in regulating the observed gene expression. To rationally elucidate this, the relative contributions of the three factors were ranked and the differentially expressed genes (adjusted *P* < 0.01) with the same order of factor contributions were grouped together (Fig. 2A).

The promoter regions of each group of genes were queried for overrepresentation of motifs using DREME (15). From the same genes, overrepresentations of GO terms were identified with gene-set enrichment analysis. This revealed the involvement of several transcription factors orchestrating the expressional changes (Fig. 2B). For instance, motif A is seemingly involved in the upregulation of genes upon nitrogen limitation, while a further upregulation is observed when *DGA1* is overexpressed (group 1). Genes that show this behavior are enriched for involvement in autophagy. Notably, Mig1-like motifs (D and H) were identified in genes strongly downregulated upon nitrogen limitation. These genes are, among others, enriched for beta-oxidation, corroborating previous identifications of this motif from batch fermentations (6). Downregulation of amino acid biosynthesis under conditions of the DGA1 × N-*lim* interaction (Fig. 1C; Fig. 2A, group 4) was potentially regulated through transcription factors with motifs (E and G) that bear resemblance to *S. cerevisiae* Gcn4 and Leu3 motifs. Contrastingly, Gln3-like motifs B and F were also found in group 2, where the DGA1 × N-*lim* interaction had very little effect on the transcript levels, while nitrogen limitation had a strong positive effect. While this agrees with the strong upregulation of genes involved in autophagy and allantoin degradation, motif A has an additional positive impact on genes involved in autophagy upon DGA1 × N-*lim* interaction (Fig. 1C).

### Correlating transcript and protein levels.

Changes in transcript levels can be interpreted as representing attempts to adapt to changing conditions, especially if these changes are orchestrated through shared transcription factors. However, changes in transcripts are not necessarily translated into changes in the levels of proteins. We therefore performed relative quantifications of proteins using the accurate mass and time (AMT) tag proteomics approach (16). Normalized peptide abundances obtained from the proteomics analysis were subsequently compared with normalized read counts obtained from RNAseq. To increase the confidence of the correlation between protein and transcripts, we focused only on the genes for which data were obtained from all 12 samples by both RNAseq and proteomics. This subset consisted of 1,516 genes, corresponding to 21.1% of the 7,170 genes annotated in the *Y. lipolytica* genome.

**FIG 3:**
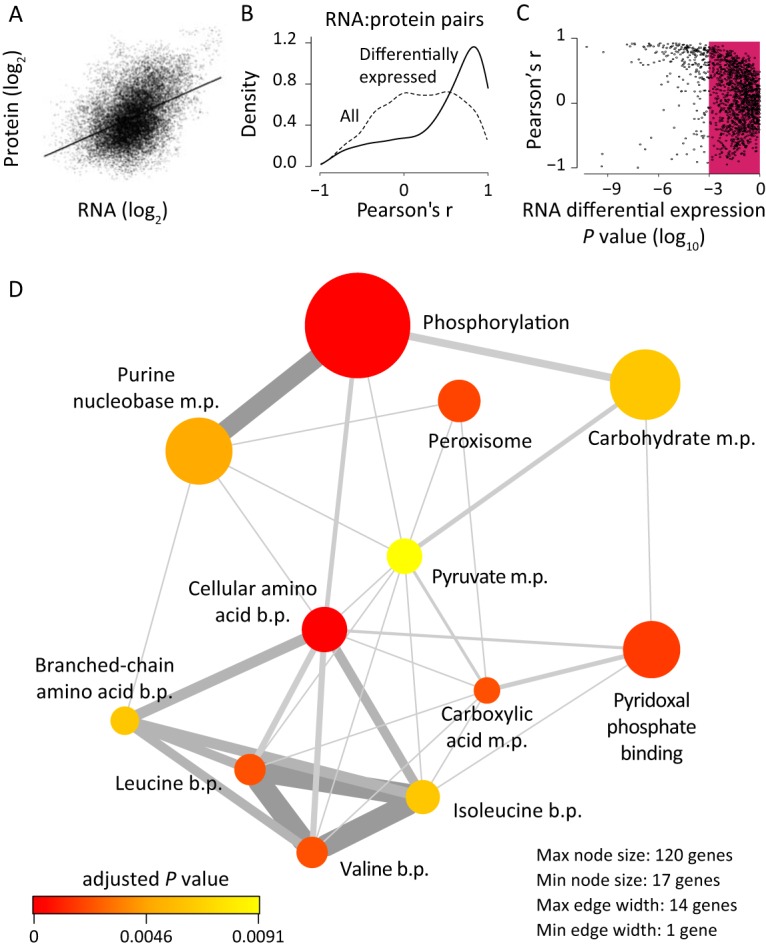
Correlation of log-normalized RNAseq read counts with log-normalized protein counts. (A) All measured RNA and protein counts combined in one comparison, with poor correlation observed. (B) Density plot of Pearson’s *r* for all RNA-protein pairs (dotted line) and for only those RNA-protein pairs that showed differential expression at the level of RNA (adjusted *P* < 0.01; solid line). (C) Representation of the correlations of both the genes that were not significantly changed (in red box) and the genes whose transcripts were found to have changed in comparisons of all chemostats (adjusted *P* < 0.01). (D) Network plot of enriched GO terms in the highly correlated gene pairs (*P* < 0.01). The size of the node represents the number of genes within the GO term. The node’s color represents the adjusted *P* value. The thickness of the edge indicates the number of genes overlapping two GO terms. m.p., metabolic process; b.p., biosynthetic process.

The correlation between all transcript-protein pairs was low (Pearson’s *r*, 0.426) (Fig. 3A). This would be expected due to the differences in the translation rates for each protein (reviewed in reference 17), while low correlations are also expected for non-differentially expressed genes. Instead, when each transcript was correlated with its corresponding protein across conditions, the correlation over all 12 experiments was moderately enriched for positive correlations between transcript and protein (Fig. 3B). Separating 444 differentially expressed transcript-protein pairs (selected from the general linear model at the transcript level, adjusted *P* < 0.01; Data Set S1) resulted in a strong enrichment for positive correlation (Fig. 3B; Data Set S2). Additionally, there seems to be a trend toward stronger correlations for genes with a greater level of significant differential expression (Fig. 3C). It is worth noting that autophagy was upregulated through the DGA1 × N-*lim* factor, while ribosomes were (moderately) upregulated due to *DGA1* overexpression (Fig. 1). These changes are likely to affect protein half-lives and translation rates, modulating the protein concentration by means other than transcript levels. Nonetheless, the proteomics data supported the changes identified from the general linear model, as a hypergeometric gene-set enrichment analysis of highly correlated genes (*P* < 0.01) unveiled similar GO terms (Fig. 3D).

10.1128/mBio.00857-17.9 DATA SET S2  Correlation between RNA and protein levels. An Excel file with differentially expressed genes (adjusted *P* < 0.05), as identified from the general linear model, that has data from both RNAseq and proteomics is shown. Correlation is indicated by Pearson’s *r* and the associated *P* value. For each gene, predicted GO terms are indicated, as obtained from reference 11. Download DATA SET S2, XLSX file, 0.04 MB. Copyright © 2017 Kerkhoven et al. 2017 Kerkhoven et al. This content is distributed under the terms of the Creative Commons Attribution 4.0 International license.

### Control of fluxes.

The combination of transcriptomics, proteomics, and binding-motif searches corroborates that amino acid biosynthesis and beta-oxidation are transcriptionally regulated, giving rise to the issue of whether these changes are also reflected in changes of metabolic fluxes. We therefore calculated metabolic fluxes using a genome-scale metabolic model by constraining measured exchange fluxes (Fig. S1; Table 1) and using random sampling of the solution space (18). This analysis was performed through four cross-comparisons: nitrogen versus carbon restriction in WT and *DGA1* strains and *DGA1* versus WT strains during nitrogen and carbon limitation. For each flux-gene pair, correlations were calculated from *Z* scores indicating the observed changes in metabolic flux and transcript and protein levels.

The number of strongly correlating fluxes differed drastically between the different comparisons (see Table S1 in the supplemental material). No large changes are expected in fluxes when phenotypic changes are only moderate. With only a few exchange fluxes constraining the intracellular fluxes, the random sampling approach gives a relatively high average relative standard deviation (RSD) for all flux-carrying reactions in the four conditions, and the fluxes therefore must change drastically for a significant *Z* score (typically >2) to occur. This approach is therefore likely to give an underrepresentation of fluxes that are correlated with changes in transcripts and protein levels.

10.1128/mBio.00857-17.7 TABLE S1  Correlations between flux, protein, and RNA. Download TABLE S1, DOCX file, 0.02 MB. Copyright © 2017 Kerkhoven et al. 2017 Kerkhoven et al. This content is distributed under the terms of the Creative Commons Attribution 4.0 International license.

**FIG 4:**
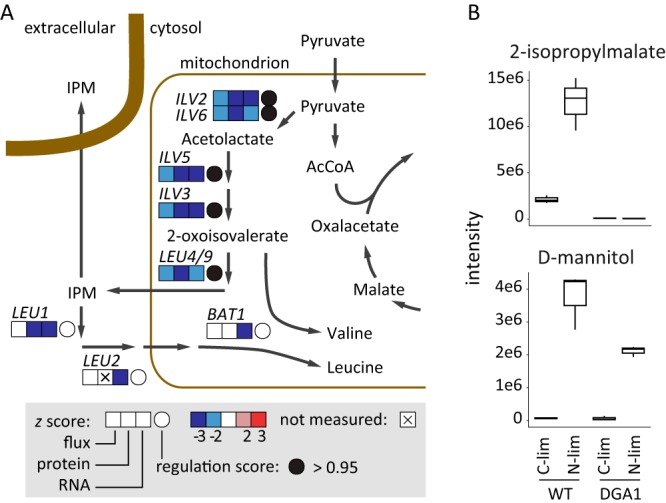
Regulation of *Y. lipolytica* metabolism. (A) Transcriptional downregulation of leucine biosynthesis. The boxes indicate *z* scores, incorporating both fold change and significance values. Regulation scores indicate correlations between all three levels and are calculated as detailed in Materials and Methods. (B) Relative intensity levels of two metabolites as measured by exometabolomics (also represented in Fig. S3 in the supplemental material). While most carbohydrates (e.g., d-mannitol) are excreted during nitrogen limitation in both WT and *DGA1* strains, 2-isopropylmalate (IPM) is no longer excreted when nitrogen limitation co-occurs with *DGA1* overexpression.

The overall observation is that transcriptionally regulated fluxes are downregulated. In each comparison, the reference condition had a lower lipid yield, and increased lipid accumulation was not associated with an upregulation of lipid biosynthetic enzymes. The highest numbers of transcriptionally regulated fluxes were seen in the comparison of nitrogen limitation to carbon limitation with the *DGA1* overexpression strain, a result which can be expected from the changes in environmental conditions and lipid phenotype. In the comparison of the *DGA1* overexpression strain to the control strain during nitrogen limitation, only a few reactions were transcriptionally regulated. Interestingly, most transcriptionally regulated genes were those involved in leucine biosynthesis (Fig. 4A), correlating the observed transcriptional changes with not only protein levels but also changes in metabolic fluxes.

10.1128/mBio.00857-17.3 FIG S3  Contributions of different factors to levels of intracellular and extracellular metabolites. Whether each of the three factors from the general linear model significantly influenced its level is indicated per metabolite (directional adjusted *P* values). The surprising decrease in the 2-isopropylmalate level seen under conditions in which nitrogen limitation co-occurred with *DGA1* overexpression is noteworthy (see also Fig. 4C). 2-Isopropylmalate and leucine are highlighted. Download FIG S3, PDF file, 3.4 MB. Copyright © 2017 Kerkhoven et al. 2017 Kerkhoven et al. This content is distributed under the terms of the Creative Commons Attribution 4.0 International license.

### Metabolome analysis.

To obtain further insight into whether flux changes are associated with changes in metabolite levels, we performed untargeted metabolomics analysis of intracellular and extracellular metabolites. Linear modeling was used to separate the changes in relative metabolite levels, and this analysis showed that overexpression of *DGA1* had limited effects on metabolite levels (Fig. S3 to S5). The main differences in extracellular metabolites were the excretion of sugars, sugar alcohols, and disaccharides under nitrogen restriction conditions. During batch stage, *Y. lipolytica* excretes disaccharides as an overflow metabolite when glucose is provided in excess, while the disaccharides are subsequently consumed upon glucose depletion (19). One of the metabolites excreted upon nitrogen restriction is the leucine biosynthetic pathway intermediate 2-isopropylmalate (IPM). However, the simultaneous overexpression of *DGA1* resulted in decreased excretion of this metabolite (Fig. 4B), even while the DGA1 × N-*lim* interaction did not significantly influence the excretion of the other carbohydrates (Fig. S3). This lack of IPM excretion is consistent with the transcriptional downregulation of the leucine biosynthetic pathway under the conditions with the highest lipid accumulation, likely through binding of a Leu3-like transcription factor to the leucine biosynthetic genes (cf. group 4 in Fig. 2A; Fig. 4A).

10.1128/mBio.00857-17.4 FIG S4  Exometabolomics signal intensities at all conditions. For each metabolite, its relative log-normalized signal intensities from GC-MS analysis are indicated for each of the four conditions. Download FIG S4, PDF file, 0.1 MB. Copyright © 2017 Kerkhoven et al. 2017 Kerkhoven et al. This content is distributed under the terms of the Creative Commons Attribution 4.0 International license.

10.1128/mBio.00857-17.5 FIG S5  Endometabolomics signal intensities at all conditions. For each metabolite, its relative log-normalized signal intensities from GC-MS analysis is indicated for each of the four conditions. Download FIG S5, PDF file, 0.1 MB. Copyright © 2017 Kerkhoven et al. 2017 Kerkhoven et al. This content is distributed under the terms of the Creative Commons Attribution 4.0 International license.

The changes in intracellular metabolite levels were more difficult to decipher. As seen with the external metabolites, nitrogen limitation resulted in increased levels of carbohydrates and organic acids such as glycolysis and tricarboxylic acid (TCA) cycle intermediates (Fig. S3). Additional *DGA1* overexpression increases the levels of many of the same metabolites. The increase in the levels of many amino acids resulting from DGA1 × N-*lim* interaction, even though amino acid biosynthetic pathways were downregulated, is surprising. The levels of leucine and valine also increased, while branched-chain amino acid biosynthesis was strongly downregulated at the level of transcription. As autophagy is strongly upregulated through DGA1 × N-*lim* interactions, it is possible that the increased amino acid levels were a consequence of protein degradation occurring through autophagy rather than as a consequence of increased *de novo* biosynthesis. These elevated levels could result in downregulation of amino acid biosynthesis, as particularly seen for the branched-chain amino acid biosynthesis. A cell undergoing nitrogen limitation would potentially benefit from increased expression of branched-chain amino acid transaminases (BCAT), as a means of shuffling amino groups between the products of protein degradation. Nonetheless, BCAT genes *BAT1* and *BAT2* respond differently to nitrogen limitation (Fig. S6). While cytosolic *BAT2* is upregulated irrespective of whether or not *DGA1* overexpression is present, mitochondrial *BAT1* instead shows an expression response similar to that seen with, e.g., leucine biosynthesis. This suggests that the primary function of *BAT1* is *de novo* biosynthesis, while *BAT2* is instead involved in reshuffling amino groups within the cytosolic branched-chain amino acid pool. The observed amino acid levels, however, do not provide a clear picture supporting this remodeling of amino acid metabolism.

10.1128/mBio.00857-17.6 FIG S6  Expression levels of *BAT1* and *BAT2*. The two branched-chain amino acid transaminases show different transcriptional responses upon nitrogen limitation in a *DGA1*-overexpressing strain. Download FIG S6, PDF file, 3.4 MB. Copyright © 2017 Kerkhoven et al. 2017 Kerkhoven et al. This content is distributed under the terms of the Creative Commons Attribution 4.0 International license.

## DISCUSSION

Lipid accumulation in *Y. lipolytica* is a complex trait where both genetic and environmental factors have influence. In this study, we observed clear phenotypical changes on the level of lipid accumulation, induced by both genetic factors and environmental factors, and we have linked these phenotypic changes to regulatory changes.

As postulated previously, lipid accumulation is not achieved through transcriptional regulation of lipid biosynthetic enzymes (6, 7, 11), while it is conceivable that posttranslational modifications play a role (6). Instead, nitrogen limitation induces a transcriptional downregulation of beta-oxidation, thereby positively affecting the lipid levels. We found that this downregulation may be regulated by Mig1 (Fig. 2B), and, as beta-oxidation is upregulated in *snf1Δ* and *mig1Δ* strains (8, 9), it is likely that binding of Mig1 represses expression of beta-oxidation genes. ATP-citrate lyase (ACL), malic enzyme, oxoglutarate dehydrogenase, and glycerol 3-phosphate dehydrogenase genes are also upregulated in a *mig1Δ* strain, and most of these genes were found here to be downregulated in nitrogen-limited chemostats. Surprisingly, transcripts of the two ACL subunits were significantly downregulated whereas protein levels were substantially upregulated, indicating complex regulation of this protein.

In our multifactorial analysis, we found a clear downregulation of amino acid biosynthetic pathways due to an interaction between *DGA1* overexpression and nitrogen restriction. This downregulation was not an effect of nitrogen limitation by itself, as observed previously (11), but was observed only as an interaction of nitrogen limitation with *DGA1* overexpression (see Fig. S2 in the supplemental material). The pathway most strongly affected by the DGA1 × N-*lim* interaction was leucine biosynthesis, supported not only by RNAseq data but also by proteomics data, as well as by estimation of fluxes and metabolomics data.

**FIG 5:**
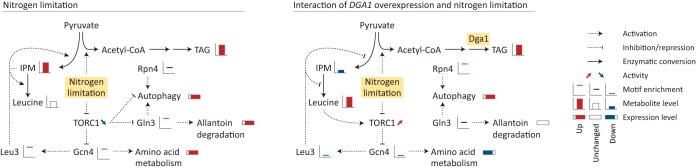
Schematic overview of responses to either nitrogen limitation or DGA1 × N-*lim* interaction. Potential regulatory networks are drawn, guided by changes in motif enrichments and in metabolite and expression levels. Leucine activates TORC1 only as a response to DGA1 × N-*lim* interaction, as leucine levels are not increased in the presence of nitrogen limitation alone. Gln3 and autophagy are seemingly not repressed by TORC1 as a response to DGA1 × N-*lim* interaction. Leu3 either represses or activates expression of leucine biosynthesis, depending on IPM levels. While TORC1 is repressed upon nitrogen limitation, its activity is seemingly modulated upon simultaneous *DGA1* overexpression, likely due to the sensing of increased leucine levels, mediated by leucyl-tRNA synthetase.

The leucine biosynthetic genes are furthermore regulated by a *S. cerevisiae* Leu3-like transcription factor. In *S. cerevisiae*, Leu3 inhibits the expression of the *ILV* and *LEU* genes of branched-chain amino acid biosynthesis, except under conditions of isopropylmalate (IPM) accumulation, which is indicative of leucine depletion (20). From the combined data, a picture emerges where IPM plays an important role in the regulatory responses to different factors, where two different regulatory states both result in lipid accumulation (Fig. 5). Nitrogen limitation by itself enhances lipid accumulation by increasing the flux through acetyl-CoA. Nitrogen limitation also results in repression of TORC1, and it has been reported that TORC1 inhibition by sirolimus increases lipid accumulation (4). Among other effects, TORC1 inhibition results in activation of Gln3, Gcn4, and Leu3 (Fig. 5). This is apparent from searches of the binding motifs, where nitrogen limitation makes a strong positive contribution to the expression levels of group 2 and group 4, which are enriched for nitrogen utilization and amino acid biosynthesis (Fig. 2). Upon nitrogen limitation, IPM levels increase (Fig. S3), and the interaction of IPM with Leu3 further activates the expression of *ILV* and *LEU* genes (shown for *ILV3* in Fig. 1C). The interaction of *DGA1* overexpression and nitrogen limitation has a very different regulatory effect (Fig. 5). How this behavior is invoked remains unclear, but it is conceivable that *DGA1* overexpression enforced a larger redistribution of the pyruvate flux, toward lipid instead of leucine biosynthesis. This reduced the level of IPM (Fig. S3), and Leu3 then acted as a repressor of *ILV* and *LEU* genes (Fig. 1C). Simultaneously, leucine levels increased (Fig. S3), and an additional regulatory pathway then came into play. In *S. cerevisiae*, leucyl-tRNA synthetase (LRS) can activate TORC1 by phosphorylation through interactions with Gtr1 in a leucine-dependent manner (21). Furthermore, supplementation of more leucine to a leucine-auxotrophic *Y. lipolytica* resulted in higher lipid accumulation (4). The increased leucine level is potentially activating TORC1, and while nitrogen limitation continues to inhibit TORC1 activity, it seems that the leucine signal is stronger. The activated TORC1 then represses Gcn4 and Leu3, an effect which presents itself in the form of strong downregulation of various amino acid biosynthetic pathways (Fig. 1B), effectively causing a positive-feedback loop. While TORC1 is also able to repress Gln3 and autophagy, it seems that this does not happen through the DGA1 × N-*lim* interaction but potentially through an alternative mechanism.

While nitrogen limitation, or sirolimus treatment, represses TORC1 and induces lipid accumulation, we observed the highest lipid accumulation when TORC1 activity was modulated by leucine levels. It seems that this regulatory behavior is active only after a certain lipid accumulation threshold is reached, as only the interaction of *DGA1* overexpression and nitrogen limitation unveiled this regulation.

Our results indicate the involvement of leucine biosynthesis in the seemingly unrelated phenotype of lipid accumulation in *Y. lipolytica*. Previously, leucine has been implicated in other complex phenotypes, e.g., ethanol tolerance in *S. cerevisiae* (22). Jointly, these findings show that leucine functions as a key metabolic node in diverse phenotypes across yeast species.

## MATERIALS AND METHODS

### Yeast strains, cultivations, and lipid analysis.

The *Y. lipolytica* strains used in this study were DGA1 overexpression strain MTYL053 and the corresponding MTYL038 control strain, both of which are prototrophic strains derived from Po1g as described in reference 2. Chemostat cultivations were performed at a dilution rate of 0.05 h^−1^ at 30°C in 1.2-liter bioreactors (DASGIP, Jülich, Germany) with a working volume of 750 ml at pH 3.5, controlled with 2 M KOH. A stirring rate of 600 rpm and an airflow rate of 1 vessel volume per min (vvm) kept the concentration of dissolved oxygen above 30%. For carbon restriction experiments, 1 liter of medium contained 5 g glucose, 5 g (NH_4_)_2_SO_4_, 3 g KH_2_PO_4_, 0.5 g MgSO_4_ ⋅ 7 H_2_O, vitamins and trace metal solutions (23), and 125 μl Antifoam 204 (Sigma-Aldrich, St. Louis, MO, USA). A similar medium was used for nitrogen restriction experiments where the glucose level was increased to 25 g and the (NH_4_)_2_SO_4_ level was decreased to 0.5 g while the SO_4_ levels were kept constant by adding 5.96 g K_2_SO_4_ ⋅ CO_2_, and residual O_2_ levels in the exhaust gas were measured by the use of an online gas analyzer (DASGIP). Samples for various analyses were rapidly taken at the steady state, defined as stable CO_2_ and O_2_ outflow and optical density. Lipid content and fatty acid composition were analyzed using gas chromatography-mass spectrometry (GC-MS) and liquid chromatography-charged aerosol detection (LC-CAD), respectively, as described previously (24, 25), and data were normalized to dry cell weight. Fermentation and lipid and fatty acid data for the DGA1 strain were obtained from reference 11.

### RNAseq analysis.

For RNA analysis, samples were rapidly taken from steady-state chemostats and stored at −80°C. Total RNA was extracted using Trizol (Invitrogen, Carlsbad, CA) and a FastPrep homogenizer (MP Biomedicals, Santa Ana, CA, USA) with 1-mm-diameter silica beads. Further RNA preparation and RNAseq were performed by SciLifeLab in Uppsala, Sweden, on their IonTorrent platform. Data from the control strain have been deposited at ArrayExpress (http://www.ebi.ac.uk/arrayexpress/experiments/E-MTAB-5284/), while data for the DGA1 strain are available from http://www.ebi.ac.uk/arrayexpress/experiments/E-MTAB-3837/. RNAseq reads were mapped to the *Y. lipolytica* CLIB122 reference genome with Bowtie 2.1.0 (26) and counted with HTseq (27). Transcripts with at least 3 libraries with more than 1 cpm were normalized using the trimmed means of M values (TMM) (28) and transformed using voom (29). Differential gene expression was analyzed following the following general linear model: *expr* = *β*_0_ + *β_Nlim_x_Nlim_* + *β_DGA_x_DGA_* + *β*_DGA_._*Nlim*_*x*_DGA_._*Nlim*_ + *ε*. Log-transformed count data and differential expression analysis data are available at the following doi: 10.6084/m9.figshare.4990394. For studies of correlation between RNA, protein, and fluxes, normalized and log-transformed counts were used, while correlation scores were calculated according to a previously described method (18).

### Gene-set analysis and motif searching.

Gene-set analysis was performed using the Piano package (30) for R. GO terms were obtained from reference 11, while those sets corresponding to “cellular localization” and gene sets with fewer than 5 or more than 500 genes were discarded. Consensus gene-set analysis was performed in a routine manner, and a rank score cutoff value of 1 was used for visualization. For motif searching, genes with an adjusted *P* value of <0.01 were manually sorted by ranking the coefficients of the three experimental factors in the general linear model (see Fig. 2A). Motif searches were performed using DREME (15), with the genes in each cluster as primary sequences, all other genes as control sequences, and an *E* value cutoff value of 10^−3^. Obtained motifs were queried for similarity to *S. cerevisiae* motifs using Tomtom (31).

### Proteomics.

Total cell protein was obtained via a chloroform/methanol-water extraction method that provides discrete protein, metabolite, and lipid fractions (32) and was digested into peptides as described in reference 6. Label-free quantitative proteomics data were obtained using the accurate mass and time (AMT) tag approach (reviewed in reference 16). Briefly, aliquots of each sample were pooled equally, fractioned into 48 fractions, and analyzed by LC-tandem MS (LC-MS/MS) to establish an AMT tag database of identified peptides and proteins, also described in reference 6. Subsequently, each sample was analyzed separately without fractionation, the masses and normalized LC retention times of detected peptides were compared to entries in the AMT tag database for identification, and peptides were quantified using the integrated LC-MS peak as also described in reference 6. Quality control processing removed peptides with an insufficient amount of data across all samples (33), while peptide abundance values within a sample were normalized using a rank invariant peptide selection approach (33). Protein level abundances were estimated using a standard reference-based methodology, R-Rollup (34). For further differential expression analysis and to facilitate comparison with transcript counts, protein counts were normalized and transformed using the same normalization and transformation methods as are detailed for RNAseq data above. Log-transformed count data and differential expression analysis data are available at the following doi: 10.6084/m9.figshare.4990394.

### Metabolomics.

For the measurements of intracellular metabolites, 20 ml of cell suspension was passed through a 0.5 μm nylon filter and was washed with 5 ml 150 mM ammonium bicarbonate. The cells were quenched with 2 ml of cold chloroform/methanol (2:1 [vol/vol]) and 0.5 ml of H_2_O containing 0.72 mM anthranilate as an internal standard. Cell debris was pelleted at 15,000 × *g* for 10 min, and the upper phase of polar metabolites was collected and snap-frozen in liquid nitrogen before sample analysis. Extracellular metabolites were collected through a similar protocol of phase separation of the cell-free spent medium after filtration. The extracted metabolites were analyzed as previously reported (19). Briefly, extracted metabolites were completely dried *in vacuo* and subjected to chemical derivatization for GC-MS analysis. The collected data were processed, and experimental metabolite spectra and retention indices were matched to entries in an in-house version of the Agilent Fiehn metabolomics library, as well as to the NIST14 GC-MS library (35, 36) or by using spectra alone (denoted “NIST”).

### Genome-scale metabolic modeling.

Metabolic fluxes were estimated using *iYali4*, a genome-scale model of *Y. lipolytica* metabolism (11). For each experimental condition, the model was adjusted to match the lipid and fatty acid measurements (represented in the biomass composition) and the relevant exchange fluxes. For determining flux ranges, the measured exchange fluxes and growth rate were allowed to vary within 1 standard deviation from the average measured values, while non-growth-associated maintenance (NGAM) energy was allowed to vary 5% around its maximum value, determined by setting the NGAM energy parameter as the objective function. Subsequent random sampling of these models delivered the *Z* scores, which were correlated with RNA expression data (18). The condition-specific models are available at the following doi: 10.6084/m9.figshare.4990394.

## References

[1] Ledesma-Amaro R, Nicaud JM 2016 Yarrowia lipolytica as a biotechnological chassis to produce usual and unusual fatty acids. Prog Lipid Res 61:40–50. doi:10.1016/j.plipres.2015.12.001. 26703186

[2] Tai M, Stephanopoulos G 2013 Engineering the push and pull of lipid biosynthesis in oleaginous yeast Yarrowia lipolytica for biofuel production. Metab Eng 15:1–9. doi:10.1016/j.ymben.2012.08.007. 23026119

[3] Qiao K, Imam Abidi SH, Liu H, Zhang H, Chakraborty S, Watson N, Kumaran Ajikumar P, Stephanopoulos G 2015 Engineering lipid overproduction in the oleaginous yeast Yarrowia lipolytica. Metab Eng 29:56–65. doi:10.1016/j.ymben.2015.02.005. 25732624

[4] Blazeck J, Hill A, Liu L, Knight R, Miller J, Pan A, Otoupal P, Alper HS 2014 Harnessing Yarrowia lipolytica lipogenesis to create a platform for lipid and biofuel production. Nat Commun 5:3131. doi:10.1038/ncomms4131. 24445655

[5] Liu L, Markham K, Blazeck J, Zhou N, Leon D, Otoupal P, Alper HS 2015 Surveying the lipogenesis landscape in Yarrowia lipolytica through understanding the function of a Mga2p regulatory protein mutant. Metab Eng 31:102–111. doi:10.1016/j.ymben.2015.07.004. 26219673

[6] Pomraning KR, Kim YM, Nicora CD, Chu RK, Bredeweg EL, Purvine SO, Hu D, Metz TO, Baker SE 2016 Multi-omics analysis reveals regulators of the response to nitrogen limitation in Yarrowia lipolytica. BMC Genomics 17:138. doi:10.1186/s12864-016-2471-2. 26911370 PMC4766638

[7] Morin N, Cescut J, Beopoulos A, Lelandais G, Le Berre V, Uribelarrea JL, Molina-Jouve C, Nicaud JM 2011 Transcriptomic analyses during the transition from biomass production to lipid accumulation in the oleaginous yeast Yarrowia lipolytica. PLoS One 6:e27966. doi:10.1371/journal.pone.0027966. 22132183 PMC3222671

[8] Seip J, Jackson R, He H, Zhu Q, Hong SP 2013 Snf1 is a regulator of lipid accumulation in Yarrowia lipolytica. Appl Environ Microbiol 79:7360–7370. doi:10.1128/AEM.02079-13. 24056466 PMC3837744

[9] Wang ZP, Xu HM, Wang GY, Chi Z-M, Chi ZM 2013 Disruption of the MIG1 gene enhances lipid biosynthesis in the oleaginous yeast Yarrowia lipolytica ACA-DC 50109. Biochim Biophys Acta 1831:675–682. doi:10.1016/j.bbalip.2012.12.010. 23274237

[10] Beopoulos A, Haddouche R, Kabran P, Dulermo T, Chardot T, Nicaud JM 2012 Identification and characterization of DGA2, an acyltransferase of the DGAT1 acyl-CoA:diacylglycerol acyltransferase family in the oleaginous yeast Yarrowia lipolytica. New insights into the storage lipid metabolism of oleaginous yeasts. Appl Microbiol Biotechnol 93:1523–1537. doi:10.1007/s00253-011-3506-x. 21808970 PMC3275733

[11] Kerkhoven EJ, Pomraning KR, Baker SE, Nielsen J 2016 Regulation of amino-acid metabolism controls flux to lipid accumulation in Yarrowia lipolytica. npj Syst Biol Appl 2:16005. doi:10.1038/npjsba.2016.5. PMC5516929 28725468

[12] Zaman S, Lippman SI, Zhao X, Broach JR 2008 How saccharomyces responds to nutrients. Annu Rev Genet 42:27–81. doi:10.1146/annurev.genet.41.110306.130206. 18303986

[13] Bernard A, Jin M, Xu Z, Klionsky DJ 2015 A large-scale analysis of autophagy-related gene expression identifies new regulators of autophagy. Autophagy 11:2114–2122. doi:10.1080/15548627.2015.1099796. 26649943 PMC4824583

[14] Natarajan K, Meyer MR, Jackson BM, Slade D, Roberts C, Hinnebusch AG, Marton MJ 2001 Transcriptional profiling shows that Gcn4p is a master regulator of gene expression during amino acid starvation in yeast. Mol Cell Biol 21:4347–4368. doi:10.1128/MCB.21.13.4347-4368.2001. 11390663 PMC87095

[15] Bailey TL 2011 DREME: motif discovery in transcription factor ChIP-seq data. Bioinformatics 27:1653–1659. doi:10.1093/bioinformatics/btr261. 21543442 PMC3106199

[16] Zimmer JSD, Monroe ME, Qian WJ, Smith RD 2006 Advances in proteomics data analysis and display using an accurate mass and time tag approach. Mass Spectrom Rev 25:450–482. doi:10.1002/mas.20071. 16429408 PMC1829209

[17] Liu Y, Beyer A, Aebersold R 2016 On the dependency of cellular protein levels on mRNA abundance. Cell 165:535–550. doi:10.1016/j.cell.2016.03.014. 27104977

[18] Bordel S, Agren R, Nielsen J 2010 Sampling the solution space in genome-scale metabolic networks reveals transcriptional regulation in key enzymes. PLoS Comput Biol 6:e1000859. doi:10.1371/journal.pcbi.1000859. 20657658 PMC2904763

[19] Pomraning KR, Wei S, Karagiosis SA, Kim YM, Dohnalkova AC, Arey BW, Bredeweg EL, Orr G, Metz TO, Baker SE 2015 Comprehensive metabolomic, lipidomic and microscopic profiling of Yarrowia lipolytica during lipid accumulation identifies targets for increased lipogenesis. PLoS One 10:e0123188. doi:10.1371/journal.pone.0123188. 25905710 PMC4408067

[20] Kingsbury JM, Sen ND, Cardenas ME 2015 Branched-chain aminotransferases control TORC1 signaling in Saccharomyces cerevisiae. PLoS Genet 11:e1005714. doi:10.1371/journal.pgen.1005714. 26659116 PMC4684349

[21] Bonfils G, Jaquenoud M, Bontron S, Ostrowicz C, Ungermann C, De Virgilio C 2012 Leucyl-tRNA synthetase controls TORC1 via the EGO complex. Mol Cell 46:105–110. doi:10.1016/j.molcel.2012.02.009. 22424774

[22] Baerends RJS, Qiu JL, Rasmussen S, Nielsen HB, Brandt A 2009 Impaired uptake and/or utilization of leucine by Saccharomyces cerevisiae is suppressed by the SPT15-300 allele of the TATA-binding protein gene. Appl Environ Microbiol 75:6055–6061. doi:10.1128/AEM.00989-09. 19666729 PMC2753098

[23] Verduyn C, Postma E, Scheffers WA, Van Dijken JP 1992 Effect of benzoic acid on metabolic fluxes in yeasts: a continuous-culture study on the regulation of respiration and alcoholic fermentation. Yeast 8:501–517. doi:10.1002/yea.320080703. 1523884

[24] Khoomrung S, Chumnanpuen P, Jansa-Ard S, Ståhlman M, Nookaew I, Borén J, Nielsen J 2013 Rapid quantification of yeast lipid using microwave-assisted total lipid extraction and HPLC-CAD. Anal Chem 85:4912–4919. doi:10.1021/ac3032405. 23634639

[25] Khoomrung S, Chumnanpuen P, Jansa-ard S, Nookaew I, Nielsen J 2012 Fast and accurate preparation fatty acid methyl esters by microwave-assisted derivatization in the yeast Saccharomyces cerevisiae. Appl Microbiol Biotechnol 94:1637–1646. doi:10.1007/s00253-012-4125-x. 22569641

[26] Langmead B, Trapnell C, Pop M, Salzberg SL 2009 Ultrafast and memory-efficient alignment of short DNA sequences to the human genome. Genome Biol 10:R25. doi:10.1186/gb-2009-10-3-r25. 19261174 PMC2690996

[27] Anders S, Pyl PT, Huber W 2015 HTSeq—a python framework to work with high-throughput sequencing data. Bioinformatics 31:166–169. doi:10.1093/bioinformatics/btu638. 25260700 PMC4287950

[28] Robinson MD, Oshlack A 2010 A scaling normalization method for differential expression analysis of RNA-seq data. Genome Biol 11:R25. doi:10.1186/gb-2010-11-3-r25. 20196867 PMC2864565

[29] Law CW, Chen Y, Shi W, Smyth GK 2014 Voom: precision weights unlock linear model analysis tools for RNA-seq read counts. Genome Biol 15:R29. doi:10.1186/gb-2014-15-2-r29. 24485249 PMC4053721

[30] Väremo L, Nielsen J, Nookaew I 2013 Enriching the gene set analysis of genome-wide data by incorporating directionality of gene expression and combining statistical hypotheses and methods. Nucleic Acids Res 41:4378–4391. doi:10.1093/nar/gkt111. 23444143 PMC3632109

[31] Gupta S, Stamatoyannopoulos JA, Bailey TL, Noble WS 2007 Quantifying similarity between motifs. Genome Biol 8:R24. doi:10.1186/gb-2007-8-2-r24. 17324271 PMC1852410

[32] Nakayasu ES, Nicora CD, Sims AC, Burnum-Johnson KE, Kim Y, Kyle JE, Matzke MM, Shukla AK, Chu RK, Schepmoes AA, Jacobs JM, Baric RS, Webb-Robertson B-J, Smith RD, Metz TO 2016 MPLEx: a robust and universal protocol for single-sample integrative proteomic, metabolomic, and lipidomic analyses. mSystems 1:e00043-16. doi:10.1128/mSystems.00043-16. 27822525 PMC5069757

[33] Webb-Robertson BJ, Matzke MM, Jacobs JM, Pounds JG, Waters KM 2011 A statistical selection strategy for normalization procedures in LC-MS proteomics experiments through dataset-dependent ranking of normalization scaling factors. Proteomics 11:4736–4741. doi:10.1002/pmic.201100078. 22038874 PMC3517140

[34] Matzke MM, Brown JN, Gritsenko MA, Metz TO, Pounds JG, Rodland KD, Shukla AK, Smith RD, Waters KM, McDermott JE, Webb-Robertson BJ 2013 A comparative analysis of computational approaches to relative protein quantification using peptide peak intensities in label-free LC-MS proteomics experiments. Proteomics 13:493–503. doi:10.1002/pmic.201200269. 23019139 PMC3775642

[35] Kind T, Wohlgemuth G, Lee DY, Lu Y, Palazoglu M, Shahbaz S, Fiehn O 2009 FiehnLib: mass spectral and retention index libraries for metabolomics based on quadrupole and time-of-flight gas chromatography/mass spectrometry. Anal Chem 81:10038–10048. doi:10.1021/ac9019522. 19928838 PMC2805091

[36] Hiller K, Hangebrauk J, Jäger C, Spura J, Schreiber K, Schomburg D 2009 MetaboliteDetector: comprehensive analysis tool for targeted and nontargeted GC/MS based metabolome analysis. Anal Chem 81:3429–3439. doi:10.1021/ac802689c. 19358599

