# Supplementary figures and images for: Leucine Biosynthesis Is Involved in Regulating High Lipid Accumulation in Yarrowia lipolytica
Source: mBio. 2017 Jun 20;8(3):e00857-17. doi: 10.1128/mBio.00857-17 (PMC5478895; doi:10.1128/mBio.00857-17)

# Figure S1

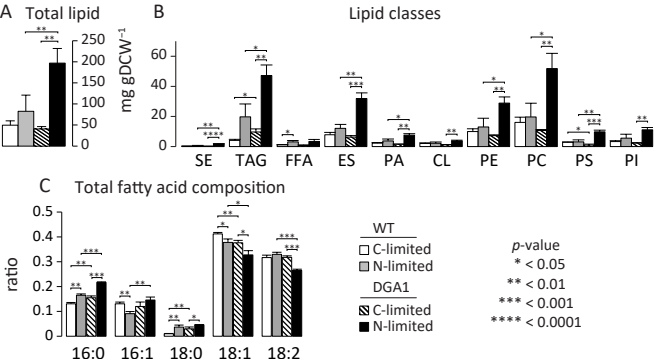

Supplement: FIG S1 [file mbo003173357sf1.pdf]

Figure S2

Normalized and log-transformed RNA read counts

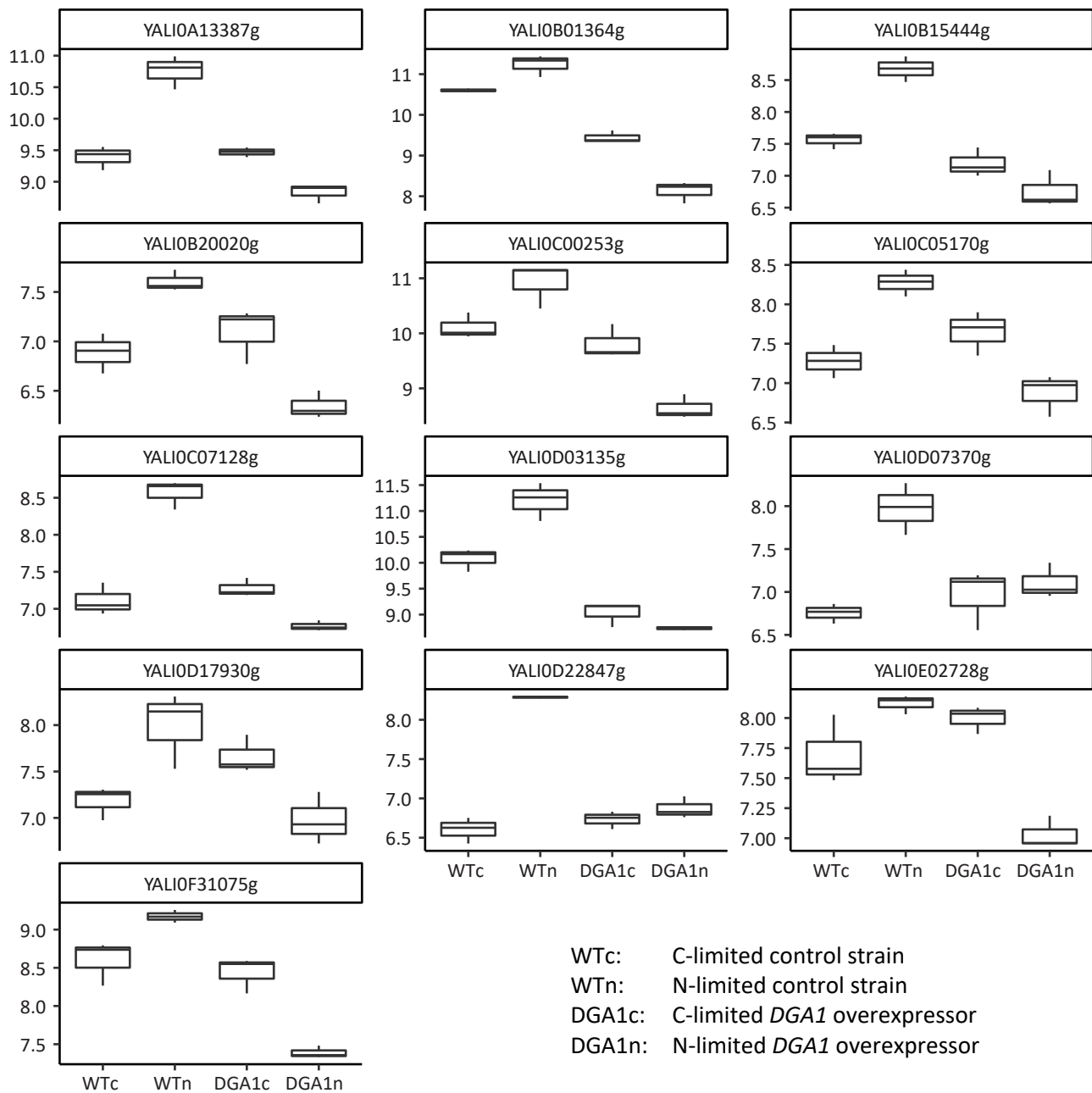

Supplement: FIG S2 [file mbo003173357sf2.pdf]

Figure S3

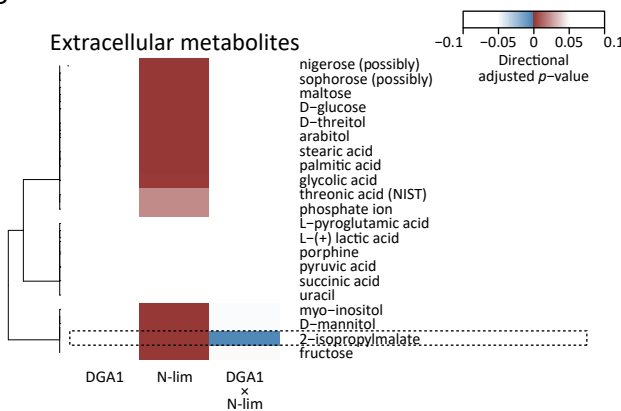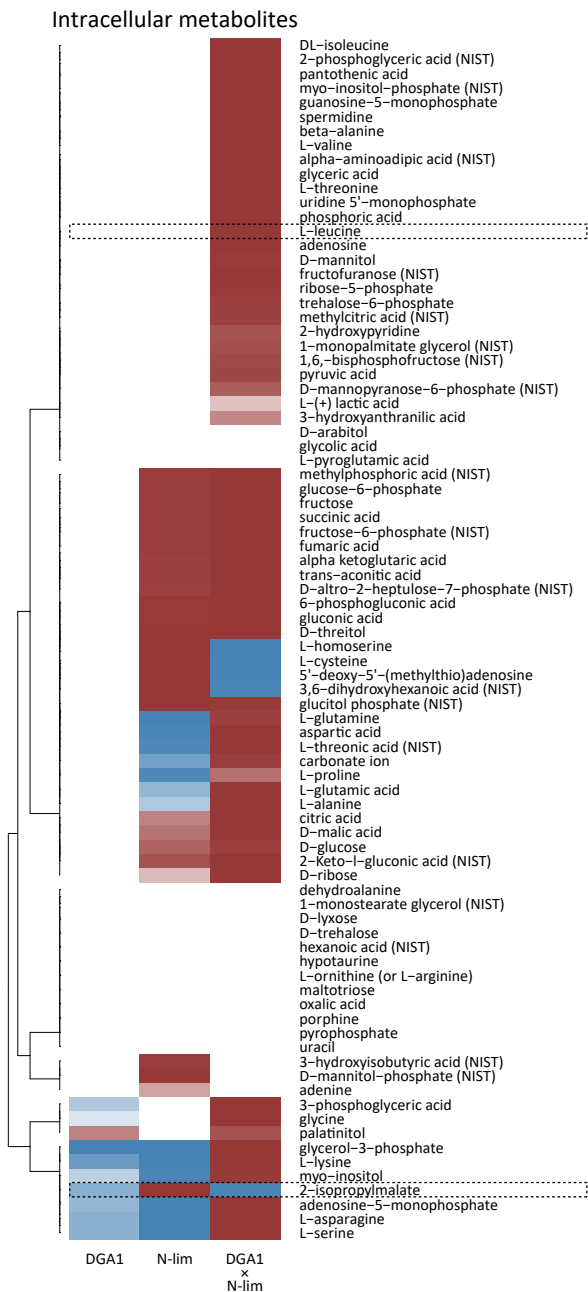

Supplement: FIG S3 [file mbo003173357sf3.pdf]

Figure S4

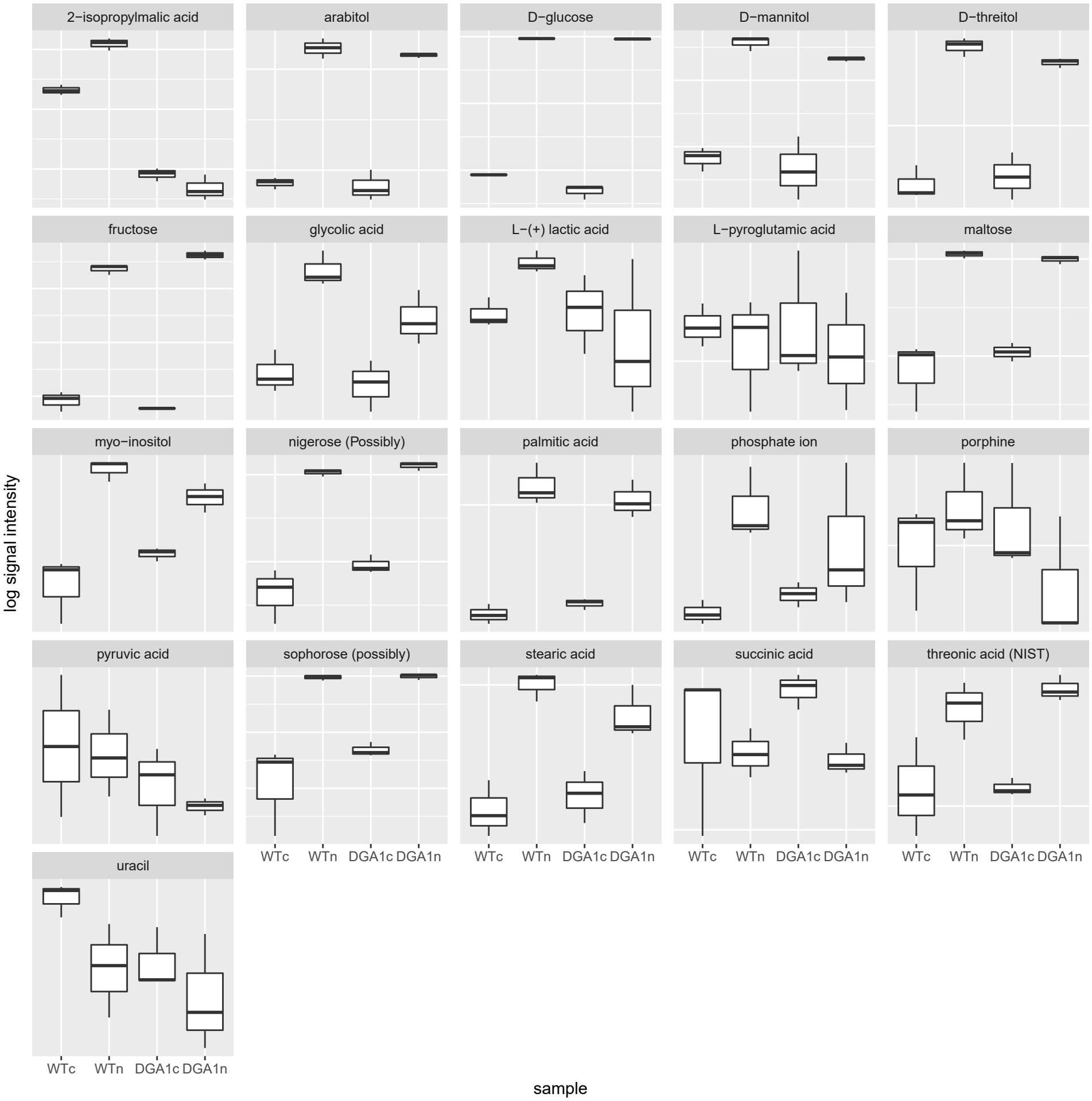

Supplement: FIG S4 [file mbo003173357sf4.pdf]

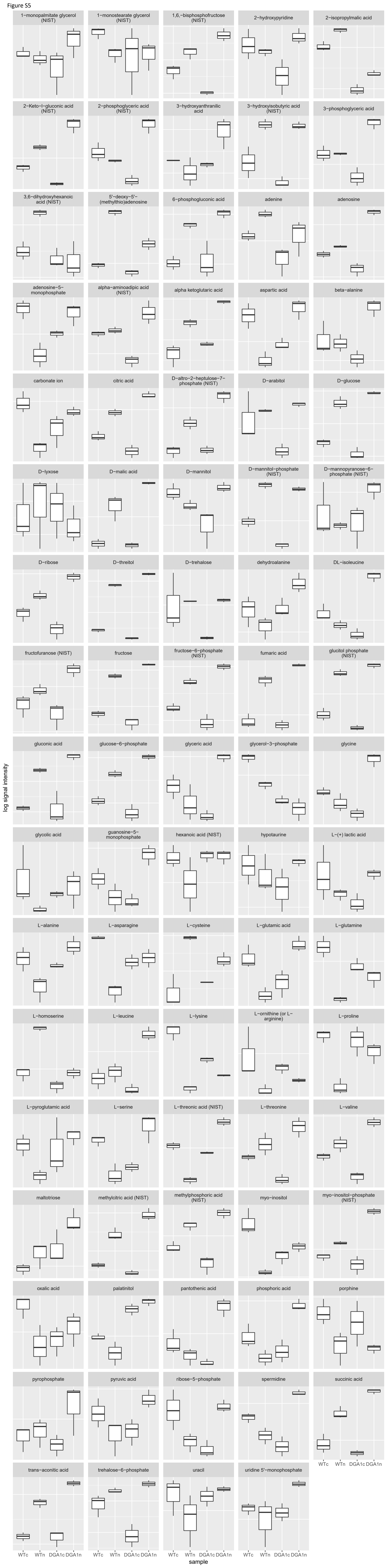

Supplement: FIG S5 [file mbo003173357sf5.pdf]

Figure S6

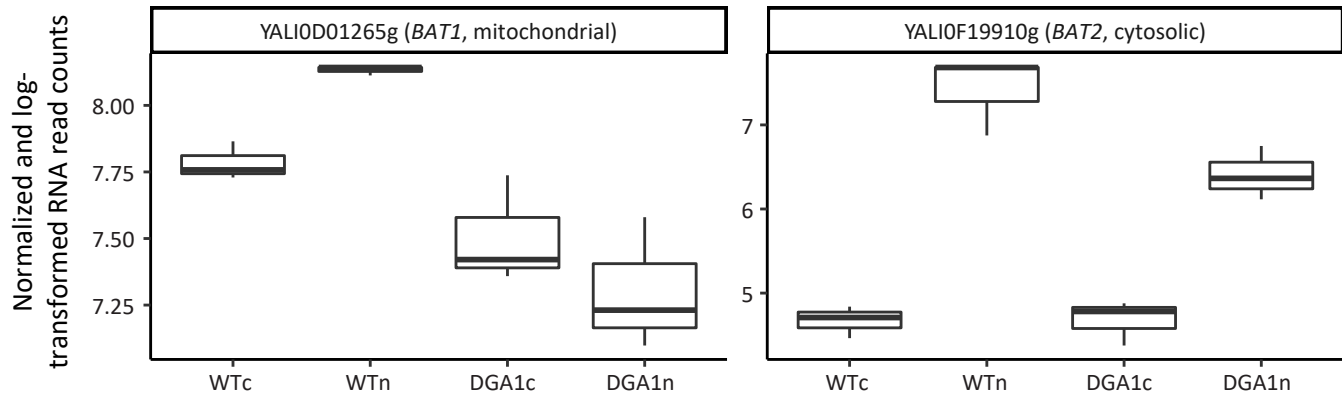

Supplement: FIG S6 [file mbo003173357sf6.pdf]
