# Supplementary material for: Leucine Biosynthesis Is Involved in Regulating High Lipid Accumulation in Yarrowia lipolytica
Source: mBio. 2017 Jun 20;8(3):e00857-17. doi: 10.1128/mBio.00857-17 (PMC5478895; doi:10.1128/mBio.00857-17)
Supplement: TABLE S1 [file mbo003173357st1.docx]

**Table S1. Correlations between flux, protein and RNA.**

| **WT N-lim vs. WT C-lim** | | | | | | | |
| --- | --- | --- | --- | --- | --- | --- | --- |
| **Gene** | **Enzyme** | **Zflux** | **Zprot** | **Zrna** | **ρProt** | **ρRNA** | **ρTR** |
| YALI0C23364g | dolichyl-phosphate-mannose--protein mannosyltransferase | -3.18 | -4.53 | -7.83 | 0.999 | 0.999 | 0.999 |
| YALI0B04312g | myo-inositol-1-phosphate synthase | 2.95 | 3.43 | 2.68 | 0.998 | 0.995 | 0.993 |
| YALI0D22022g | ATP synthase | 2.74 | 1.97 | 1.54 | 0.973 | 0.936 | 0.910 |
| YALI0F05632g | mevalonate pyrophoshate decarboxylase | 4.01 | 1.82 | 1.53 | 0.965 | 0.938 | 0.905 |
| **DGA1 N-lim vs. DGA1 C-lim** | | | | | | | |
| **Gene** | **Enzyme** | **Zflux** | **Zprot** | **Zrna** | **ρProt** | **ρRNA** | **ρTR** |
| YALI0C23408g | dihydroxy-acid dehydratase (2,3-dihydroxy-3-methylbutanoate) | -3.68 | -4.82 | -3.55 | 1.000 | 1.000 | 1.000 |
| YALI0B07447g | 2-isopropylmalate synthase | -3.53 | -7.13 | -4.38 | 1.000 | 1.000 | 1.000 |
| YALI0C23408g | dihydroxy-acid dehydratase (2,3-dihydroxy-3-methylpentanoate) | -2.97 | -4.82 | -3.55 | 0.998 | 0.998 | 0.997 |
| YALI0C23364g | dolichyl-phosphate-mannose--protein mannosyltransferase | -2.95 | -4.03 | -5.99 | 0.998 | 0.998 | 0.997 |
| YALI0F12639g^1^ | 3-dehydroquinate dehydratase | -2.95 | -6.54 | -3.77 | 0.998 | 0.998 | 0.997 |
| YALI0B01364g^1^ | 2-isopropylmalate hydratase | -2.93 | -4.92 | -4.69 | 0.998 | 0.998 | 0.997 |
| YALI0F31075g | homocitrate synthase | -2.94 | -13.6 | -3.31 | 0.998 | 0.998 | 0.996 |
| YALI0A13387g | asparagine synthase (glutamine-hydrolysing) | -2.96 | -3.13 | -4.03 | 0.998 | 0.998 | 0.996 |
| YALI0C07128g | Imidazole-glycerol-3-phosphate synthase | -2.91 | -3.08 | -3.08 | 0.997 | 0.997 | 0.994 |
| YALI0C00253g | acetolactate synthase | -3.68 | -8.44 | -2.36 | 1.000 | 0.991 | 0.991 |
| YALI0A15950g^1^ | histidinol dehydrogenase | -2.91 | -3.61 | -2.48 | 0.998 | 0.992 | 0.990 |
| YALI0E06457g | L-aminoadipate-semialdehyde dehydrogenase (NADPH) | -2.94 | -2.78 | -2.63 | 0.996 | 0.994 | 0.990 |
| YALI0C00253g | 2-aceto-2-hydroxybutanoate synthase | -2.97 | -8.44 | -2.36 | 0.998 | 0.989 | 0.988 |
| YALI0D22891g | saccharopine dehydrogenase (NADP, L-glutamate forming) | -2.94 | -5.47 | -2.11 | 0.998 | 0.981 | 0.979 |
| YALI0B20020g | 3-deoxy-D-arabino-heptulosonate 7-phosphate synthetase | -2.95 | -3.43 | -2.11 | 0.998 | 0.981 | 0.979 |
| YALI0E09493g | aspartate carbamoyltransferase | -2.94 | -4.18 | -2.06 | 0.998 | 0.979 | 0.977 |
| YALI0E16368g | sulfite reductase (NADPH2) | -2.94 | -9.66 | -1.99 | 0.998 | 0.975 | 0.973 |
| YALI0B04312g | myo-inositol-1-phosphate synthase | 49.49 | 3.37 | 1.80 | 1.000 | 0.964 | 0.963 |
| YALI0E05929g | dolichyl-phosphate-mannose--protein mannosyltransferase | -2.95 | -1.76 | -2.84 | 0.959 | 0.996 | 0.955 |
| YALI0E17479g | chorismate mutase | -2.94 | -1.64 | -4.30 | 0.948 | 0.998 | 0.946 |
| YALI0C05170g | ATP phosphoribosyltransferase | -2.91 | -1.89 | -1.93 | 0.969 | 0.971 | 0.941 |
| YALI0E02728g^1^ | 2-methylcitrate dehydratase | -2.94 | -1.55 | -5.21 | 0.938 | 0.998 | 0.936 |
| YALI0C06490g | mannose-1-phosphate guanylyltransferase | -2.95 | -2.00 | -1.69 | 0.976 | 0.953 | 0.930 |
| YALI0D17930g | chorismate synthase | -2.95 | -1.56 | -1.92 | 0.940 | 0.971 | 0.912 |
| YALI0B14399g | pyrroline-5-carboxylate reductase | -2.94 | -28.7 | -1.36 | 0.998 | 0.911 | 0.910 |
| YALI0E14751g | anthranilate synthase | -3.00 | -1.34 | -3.09 | 0.909 | 0.998 | 0.907 |
| **DGA1 C-lim vs. WT C-lim** | | | | | | | |
| **Gene** | **Enzyme** | **Zflux** | **Zprot** | **Zrna** | **ρProt** | **ρRNA** | **ρTR** |
| YALI0C06424g | glucose transport | 2.60 | 1.83 | 1.85 | 0.962 | 0.963 | 0.927 |
| **DGA1 N-lim vs. WT N-lim** | | | | | | | |
| **Gene** | **Enzyme** | **Zflux** | **Zprot** | **Zrna** | **ρProt** | **ρRNA** | **ρTR** |
| YALI0C23408g | dihydroxy-acid dehydratase (2,3-dihydroxy-3-methylbutanoate) | -2.10 | -3.90 | -8.27 | 0.982 | 0.982 | 0.964 |
| YALI0D03135g | acetohydroxy acid isomeroreductase | -2.10 | -8.81 | -3.32 | 0.982 | 0.982 | 0.964 |
| YALI0C00253g | acetolactate synthase | -2.10 | -10.9 | -3.07 | 0.982 | 0.981 | 0.963 |
| YALI0B07447g | 2-isopropylmalate synthase | -2.04 | -4.19 | -2.98 | 0.979 | 0.978 | 0.957 |
| YALI0C09636g | acetolactate synthase | -2.10 | -5.01 | -1.89 | 0.982 | 0.953 | 0.936 |
| YALI0C05951g | oleoyl-CoA desaturase, ER membrane | 21.22 | 3.62 | 1.33 | 1.000 | 0.909 | 0.909 |
| YALI0E31064g | phosphate transport | 3.48 | 3.17 | 1.30 | 0.999 | 0.904 | 0.903 |

For four comparisons, the genes with the highest cross-correlation between flux, protein and RNA changes are shown. *Z*-scores indicate direction and significant of changes, while ρProt and ρRNA are indicative of correlation between flux and either protein or RNA. ρTR indicates transcriptionally regulated genes, were RNA, protein and flux changes are all strongly correlated. A ρTR cutoff is 0.9.

^1^ Multiple reactions were annotated to these genes, with identical *Z*- and ρ-scores. Only one reaction is shown here.
